# The Relationship between Expertise in Sports, Visuospatial, and Basic Cognitive Skills

**DOI:** 10.3389/fpsyg.2016.00904

**Published:** 2016-06-17

**Authors:** Holger Heppe, Axel Kohler, Marie-Therese Fleddermann, Karen Zentgraf

**Affiliations:** ^1^Department of Human Performance and Training in Sports, Institute of Sport and Exercise Sciences, University of MünsterMünster, Germany; ^2^Otto Creutzfeldt Center for Cognitive and Behavioral Neuroscience, University of MünsterMünster, Germany; ^3^Institute of Psychology, University of MünsterMünster, Germany

**Keywords:** visuospatial performance, mental rotation, choice response time, sport expertise, basic cognitive skills

## Abstract

Team sports place high demands on visuospatial and other cognitive skills. However, there is a lack of research on visuospatial skills of elite athletes and there are heterogeneous results on basic cognitive skills of this population. Therefore, this series of studies tested different cognitive skills in elite team sports athletes. In Experiment 1, elite athletes were compared to recreational athletes, but no differences were observed between the groups in choice response time (CRT) and mental rotation (MR). To see if differences could be observed when the tested groups had a greater difference in expertise and more representative stimuli, in Experiment 2, we tested CRT and MR of elite athletes who had higher level of expertise, and we also used three-dimensional human stimuli. Overall, we still found no differences in MR; however, elite athletes did have shorter CRTs. In Experiment 3, instead of testing MR, we compared elite athletes’ and recreational athletes’ basic cognitive skills, such as processing speed, letter readout speed, memory span, and sustained attention. We found that elite athletes only performed better in sustained attention. Building on this data, in a supplementary analysis (Experiment 4) we tested whether MR and CRTs are correlated with basic cognitive skills. Results show that processing speed is the best predictor for MR, whereas letter readout speed explains most of the variance in CRTs. Finally, we discuss these findings against the backdrop of expertise and offer implications for future studies on mental rotation.

## Introduction

Over a decade ago, the first studies revealed that physical exercise may have positive transfer effects on cognitive functioning (Colcombe and Kramer, 2003; Hillman et al., 2008). The results showed that physical exercise improved cognitive performance, mainly for tasks that require greater amounts of cognitive control, but also for basic cognitive measures. Apart from physical exercise, some studies have shown that cognitive skills can also be enhanced by specific cognitive training, which applies especially for attention (Tang and Posner, 2009), but also for working memory training (Karbach and Verhaeghen, 2014). Importantly, both of these training approaches, physical exercise, and cognitive training, seem to be combined in elite sports, especially in interactive and combat sports. Imagine a midfielder in a soccer game receiving a pass when facing his own goal. In the last seconds before the pass arrives, he is attentive and checks his shoulders twice to see if a defender is behind him and to spot open spaces. When the pass arrives, he has to make a decision based on what is stored in his working memory under time pressure. This example illustrates the cognitive demands in ball sports, which include perceptive and decisive skills. Raab (2014) emphasizes the importance of both bottom-up and top-down processes involved in these decisions. Benefits in perceptual-cognitive skills at these high performance levels also seem to be feasible via representative tasks, such as video-based simulations (Broadbent et al., 2015).

Perceptual-cognitive skills in sports have traditionally been studied via the expert-performance approach using sports-related stimulation. Here, expert-athletes, near-expert athletes, and novices are confronted with visual stimuli derived from the sports domain and asked to recognize game patterns or to anticipate the direction of strokes. In a meta-analysis by Mann et al. (2007), it was shown that elite players performed better in sports-related cognitive tasks. However, this approach does not incorporate non-sport-specific stimulation and cannot characterize the transferability of the acquired skills to other domains. Yet there is some evidence that players of team sports have enhanced non-sports specific fundamental, cognitive skills which has been studied by the so-called cognitive-components approach. In their meta-analysis, Voss et al. (2010) found small-to-medium effect sizes indicating elite athletes perform better than non-elite athletes in cognitive measures, with the largest effect sizes seen for processing speed. They summarized studies that used the cognitive-components approach to investigate the relationship between sports expertise and general cognitive skills. After subdividing cognitive skills into attentional cuing, processing speed and varied attention paradigms, the authors found mixed results, but identified compelling evidence that cognitive skills can be transferred from the sports-specific to the general context. Sport type was found to be a moderator for the sport-cognition relationship, with interceptive sports (e.g., tennis or boxing) showing largest effects, followed by strategic sports, finally followed by static sports (running and swimming) showing the smallest effects. It should be noted that the distinction between non-sport-specific and sport-specific cognitive skills can be blurred. For example, in an experiment by Romeas and Faubert (2015) where subjects were tested on their ability to perceive biological motion in a virtual environment, soccer players and non-athletes were asked to identify the direction of a point-light walker. While perceiving biological motion is clearly not soccer-specific, it is essential for all interactive sports. Therefore, it is hard to draw a sharp line between sports-specific and non-sports-specific, when the transfer of cognitive skills is discussed.

There are several explanatory approaches for a positive transfer of sports on general cognitive skills; for example, it is generally known that increased aerobic capacity affects cognitive functioning (Hillman et al., 2008). In addition, coordinative challenges in sports can also be assumed to positively transfer to cognitive skills (Voelcker-Rehage et al., 2011). Marchetti et al. (2015) found evidence for a correlation between working memory updating and physical fitness. A cognitive aspect of sports games (i.e., decision making) was also suggested to be correlated to inhibition. In general, findings indicate that cognitive skills are not absolutely context-bound, and a cognitive skill transfer between different tasks can occur (Perkins and Salomon, 1989; Willis et al., 2006; Dahlin et al., 2008; Jaeggi et al., 2008).

Apart from the findings by Voss et al. (2010), further findings describe the positive relationship between cognitive skills and sports expertise. In a cross-sectional study which compared senior high-division players to low-division players and a standardized norm group, high-division players showed better executive function compared to low-division players, who, in turn, outperformed the standardized norm group (Vestberg et al., 2012). A second part of the study showed a trend to predict future success (measured by assists and goals) by executive test performance. Another soccer-related study investigated the executive functioning of highly talented and amateur youth soccer players (Verburgh et al., 2014). Highly talented players outperformed the amateur players in motor inhibition and in the ability to maintain attention. Similar results were found in a comparison of elite volleyball players and non-active controls in tests of executive control, memory, and visuospatial attention. The volleyball players performed better on executive control tasks and on a visuospatial attentional processing task, but results showed small effect sizes (Alves et al., 2013). Wang et al. (2015) also proposed better visuospatial performance of athletes when they compared female badminton players to non-athletic controls. The athletes exhibited greater task-induced modulations on neural oscillations, which were associated to better performance. Other studies have shown expertise-related advantages in distributed attention (Faubert, 2013) and a non-sport-specific, perceptual-cognitive advantage when recognizing human biological motion (Romeas and Faubert, 2015).

But there are also findings speaking against the general superiority of elite athletes in cognitive skills such as attention measures. In one study, researchers investigated whether athletes from team sports differ from non-team-sport athletes or novices in attention tasks. Participants were tested in a field-of-view test, a two-dimensional multiple object tracking test, and an inattentional blindness task (Memmert et al., 2009). Experts in team sports did not perform better in any of these attention tasks. When it comes to single response times, there are findings that suggest athletes are both superior to (Akarsu et al., 2009; Zwierko et al., 2010) and not superior to (Starkes, 1987) non-athletes. Specifically, some evidence in favor of athletes’ superior response times indicates that athletes have faster signal transmission in visual pathways, leading to shorter response times for athletes compared to controls (Zwierko et al., 2010). However, when athletes’ tasks require more than simple reaction times, for example, when choices are involved, findings show that athletes have faster response times (Zwierko et al., 2010; Heirani et al., 2012; Piras et al., 2014).

While many studies have explored the transfer of physical training on basic cognitive measures and executive functioning, very few studies have investigated a transfer on visuospatial skills. In a recent meta-analysis, it was found that visuospatial skills can be trained, including through physical exercise (Uttal et al., 2013). Regarding the different subdivisions of visuospatial skills – which are spatial visualization, spatial perception and mental rotation (Linn and Petersen, 1985) – mental rotation has been the most thoroughly investigated (Shepard and Metzler, 1971). Mental rotation describes the cognitive process of turning an object in the imagination, and it is investigated by showing rotated objects to participants, who are then told to dichotomously decide if the objects are the same or different as correctly and quickly as possible. Characteristically, response times increase linearly with increasing angle disparity. This led to the assumption that the internal representation of the stimulus is structurally analogous to a visual percept, and the mental transformation of this stimulus shows a correspondence to an external object undergoing a physical rotation. When solving these kind of tasks, different processing stages are postulated: First, the object has to be identified after perceiving the stimulus. Without the proper determination of the presented stimulus type, no further processes can take place concerning the orientation of the presented object. This information is assumed to be used in order to mentally rotate the given object via the shortest route into the zero degree position to then decide whether it is mirrored or not. Based on this decision, the motor response is prepared and executed (Cooper and Shepard, 1973). The first and last described step can be assumed to be measured in response times when stimuli are non-rotated. This part of a mental rotation test can be considered as a measure of choice response times, because choice response time tests require the perception of a stimulus and an appropriate response under time pressure. However, in these experiments, the mental rotation causes some uncertainty – participants do not know if and how far the upcoming stimulus will be rotated. This leads to increased response times compared to sole choice response time paradigms (Ilan and Miller, 1994). Therefore, the generalizability of choice response times measured in mental rotation experiments is restricted.

The underlying processes of these stages in mental rotation are not yet clarified, but some experiments have attempted to examine them. Cooper and Shepard (1973) postulated a stage-based processing, but the stages should not be understood as totally distinct. At least some temporal overlap should be assumed for the processes of mental rotation and discrimination (Ruthruff and Miller, 1995). In their pioneering experiment, Shepard and Metzler (1971) presented two twisted cube figures consisting of 10 cubes to participants which were rotated in 20° decrements twisted to each other in depth or in plane. This paradigm has also been examined with other stimuli, for example, human hands (Parsons, 1987), letters (Cooper and Shepard, 1973), or polygons (Cooper, 1975). In the majority of experiments with non-abstract stimuli, response times increased with increased angle disparity, showing a trend that was not linear, but curvilinear in many cases (Parsons, 1987). This indicates different strategies based on the nature of the stimuli, such as human figures or objects.

To investigate this further, some research groups assume embodied or egocentric processing of mental rotation (Steggemann et al., 2011), especially for human-related stimuli, because the use of these stimuli often results in quicker responses (Parsons, 1987; Steggemann et al., 2011). These egocentric strategies are assumed to involve mental processes coupled with motor mechanisms (Moreau, 2012). Hence, the involvement of motor processes is also affected by the nature of the stimuli (Wexler et al., 1998). Kosslyn et al. (1998) described the underlying neural mechanisms of mental rotation of objects and human stimuli. Using positron emission tomography, they compared mental rotations of hands and of cubes. Only mental rotations of hands led to higher activation of motor areas, speaking for a different strategy compared to object rotations. Differences in brain activation when mentally rotating human stimuli were also found in other studies (Reed and Farah, 1995; Zacks et al., 2003).

In the study by Steggemann et al. (2011), athletes with and without physical overhead experience were compared in their mental rotation skills. The athletes with overhead expertise responded faster than non-experts when human stimuli were presented in back view, but not when front-view human stimuli or letters had to be rotated. Moreau (2012) compared the mental rotation performance of elite wrestlers to a control group who did no sports. The wrestlers performed better than the control group. Another wrestling-related study was conducted in form of a 10-month intervention study. A wrestling intervention (2 h per week) led to a higher performance increase in a psychometric mental rotation test compared to a comparable running intervention (Moreau et al., 2012). Another study compared open-skill athletes, closed-skill athletes and non-athletes and found differences in mental rotation between the athletes and non-athletes (Ozel et al., 2004). However, in a study which compared soccer players to non-athletes concerning mental rotation of human and abstract figures, no differences in mental rotation were observed, but the authors did find differences in response times when stimuli were not rotated (Jansen et al., 2012). These response times can be considered as 2-choice response times.

The present study follows the cognitive-components approach and therefore implemented non-sport-specific cognitive measures to evaluate expertise differences. All in all, many studies show that there are expert advantages in sport-specific perceptual-cognitive skills. However, there are less conclusive findings about if team sports expertise affects non-sports-specific cognitive measures, and within these findings, there is a lack of research on spatial cognition. In the present study, we aimed to assess the differences between experts and recreational players in mental-rotation performance and cognitive measures. Four experiments are reported: Experiments 1 and 2 investigated the relationship between expertise and spatial cognition by testing elite and recreational players in mental rotation. Experiment 3 evaluated to what degree memory span, letter readout speed, attention, and processing speed would explain the effects of the two previous experiments. Finally, Experiment 4 directly correlated the cognitive measures with mental rotation performance. This experimental approach of examining the relationship between mental rotation response times at different angles and different cognitive measures to investigate their share of variance has not been done before, and may help to reveal which cognitive mechanisms are involved in mental rotation. This study was approved by the local ethics committee and met the requirements of the Declaration of Helsinki.

## Experiment 1: Mental Rotation of Two-Dimensional Human Figures and Choice Response Times

In Experiment 1, female elite handball or soccer players were compared to a control group consisting of recreational athletes. If a regular, long-lasting involvement in team sports on a competitive basis enhances spatial cognition, we expect these groups to show a significant difference in their mental rotation performances. Based on previous findings, we also hypothesized shorter response times of athletes at an angle disparity of 0°.

### Materials and Methods

#### Participants

Thirty female elite athletes, aged 16–34 years (*M* = 23.2; *SD* = 4.1), and 30 recreational athletes (13 females), aged 16–23 (*M* = 21.7; *SD* = 1.7), participated in the experiment. In sports science, there are different definitions of expertise levels. We chose to include participants who play handball or soccer in the first or second division in Switzerland. Their training age was 7–21 years. The recreational participants participated in different sports (mostly team sports) on a regular basis.

#### Apparatus and Stimuli

**FIGURE 1:**
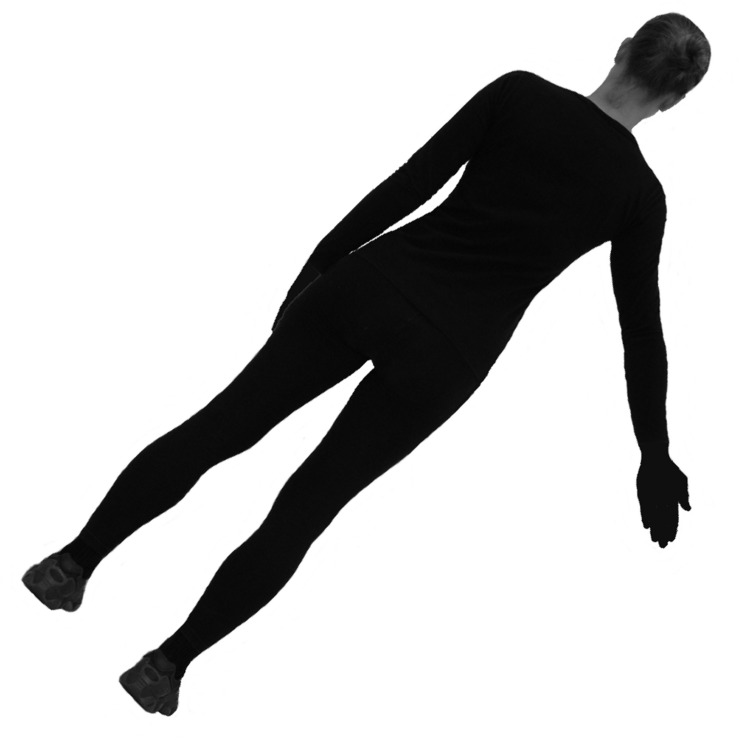
**Stimulus used in Experiment 1**. Human figure with abducted right arm, rotated 45 degrees around the depth axis.

For the presentation of the stimuli, the software Presentation^®^ of Neurobehavioral Systems (version 16.5) was used in both mental rotation experiments. Participants sat in front of a monitor and responded via two keys with their equilateral index fingers. The stimuli (**Figure 1**) consisted of human figures presented in back view, similar to the stimuli used in previous mental rotation studies (Jola and Mast, 2005; Steggemann et al., 2011). Participants had to decide as quickly and accurately as possible whether the right or left arm was outstretched. The stimuli were presented in 8 angle disparities (0°, 45°, 90°, 135°, 180°, 225°, 270°, and 315°) and two sides (left and right) and participants performed two blocks which resulted in 32 decisions (four decisions at any angle disparity). The order of stimulus presentation was randomized for each participant. When there was no significant difference, equal clock- and counterclockwise rotations were pooled.

#### Data Analysis

To test whether elite team sports athletes outperform recreational athletes in the process of encoding/giving a motor response, an independent-samples t-test was conducted for response times at the angle disparity of 0°. A repeated-measures ANOVA of all angles >0° was conducted to analyze group differences in the process of mental rotation. Response times based on wrong answers were not analyzed. Error rates were 2.62% (elite athletes) and 2.29% (recreational athletes).

### Results

**FIGURE 2:**
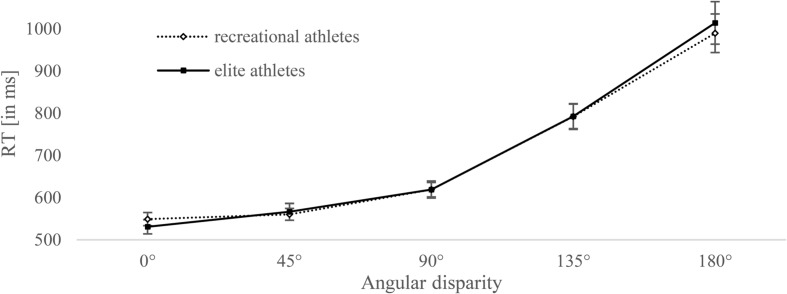
**Mean response times (RTs) for recreational and elite athletes and for each angle disparity**. Error bars represent standard errors.

**Figure 2** shows response times of both groups at the five angle disparities. There was no significant difference in response times at 0° between elite (*M* = 530.1 ms, *SD* = 92.1) and recreational athletes (*M* = 549.1 ms, *SD* = 85.4), *t*(58) = .79, *p* = 0.43, 95% CI [-0.293, 0.722], *d* = 0.22. As expected, the ANOVA with four angle disparities (45°, 90°, 135°, and 180°) showed a statistically significant main effect of disparity, *F*(3,174) = 158.7, *p* < 0.001, $\eta_{p}^{2}$ = 0.732. The main effect of group was not significant, *F*(1,58) = 0.05, *p* = 0.822, $\eta_{p}^{2}$ = 0.001; also not the interaction of disparity and group, *F*(3,174) = 0.132, *p* = 0.838, $\eta_{p}^{2}$ = 0.002.

### Discussion

We found no difference between both groups, neither in the process of mental rotation, nor in other processes (i.e., encoding and giving a motor response). This is contradictory to our hypothesis. Possibly, the different distribution of women and men in both groups could have diminished possible expertise effects. The group of recreational athletes contained more male athletes. In psychometric paper and pencil mental rotation tests, there is a stable effect of a male advantage (Peters, 2005; Voyer, 2011). However, this advantage is higher when abstract stimuli are presented instead of human stimuli (Jansen and Lehmann, 2013), as we used here. In chronometric mental rotation tests, as we did here, some findings speak for a male advantage (Voyer and Jansen, 2015), but the findings are heterogeneous. For example, one study showed a male advantage only for polygons, but none of the other stimuli (letters, animal drawings, abstract symbols, and cube figures) showed gender effects (Jansen-Osmann and Heil, 2007). Furthermore, another study showed no gender effect, neither in cube stimuli, nor in human figures (Jola and Mast, 2005). In addition, in one study which focused on cognitive skills, but not specifically on mental rotation, but on a battery of general cognitive tests, gender effects were mainly found in a physically inactive control group. They found no difference compared to participants who were physically active; thus, gender effects in cognitive tasks are assumed to be reduced by athletic experience (Alves et al., 2013). In the present experiment, the control group consisted of participants who did sports on a regular basis. A *post hoc* analysis showed that there was no significant main effect of gender, *F*(1,28) = 0.08, *p* = 0.784, $\eta_{p}^{2}$ = 0.003, and no significant interaction of gender and angle disparity, *F*(4,112) = 0.31, *p* = 0.707, $\eta_{p}^{2}$ = 0.011, within the group of recreational players. However, to control for a possible gender effect, the control group in Experiment 2 was gender-matched. According to results from Steggemann et al. (2011), we designed Experiment 2 so that it still used a two-dimensional human stimulus in back view rotated around the depth axis, but we expanded the stimuli to three dimensions: human figures were also rotated around the longitudinal axis to account for the motor and visual experiences conveyed through team sports. Instead of having the right or left arm bent, in Experiment 2, the human stimulus held a ball in its right or left hand.

Another change we made in Experiment 2 was to increase the expertise of our elite-athlete group compared to the control group (recreational athletes). There are many definitions of expertise in the research literature (Swann et al., 2015), but in this study series we defined elite athletes by the league in which they play. To raise the level of expertise in Experiment 2, we only included professional players who earn their living with their sport. Thus, athletes who took part in Experiment 2 showed more professionalism and experienced greater national competitiveness within their sport compared to the athletes in Experiment 1. Thus, the athletes in Experiment 2 can be considered, according to Swann et al. (2015), as having a higher level of expertise compared to those in Experiment 1. As in the first experiment, in Experiment 2 we hypothesized that experts outperform the controls in mental rotation performance and in choice response times.

## Experiment 2: Mental Rotation of Three-Dimensional Human Figures and Choice Response Times

### Materials and Methods

#### Participants

In this experiment, elite athletes (*n* = 27, 12 females) playing volleyball or handball were compared to recreational athletes (*n* = 27, 12 females). Mean age in the elite athletes group was 24.6 years (*SD* = 4.0, range 17–28) and in the recreational group 23.9 years (*SD* = 3.2, range 20–31). The elite group played volleyball in the first division or handball in the second division in Germany. The recreational athletes had experience in team sports. They did not perform any sports requiring frequent physical rotations or overhead positions (e.g., gymnastics).

#### Apparatus and Stimuli

**FIGURE 3:**
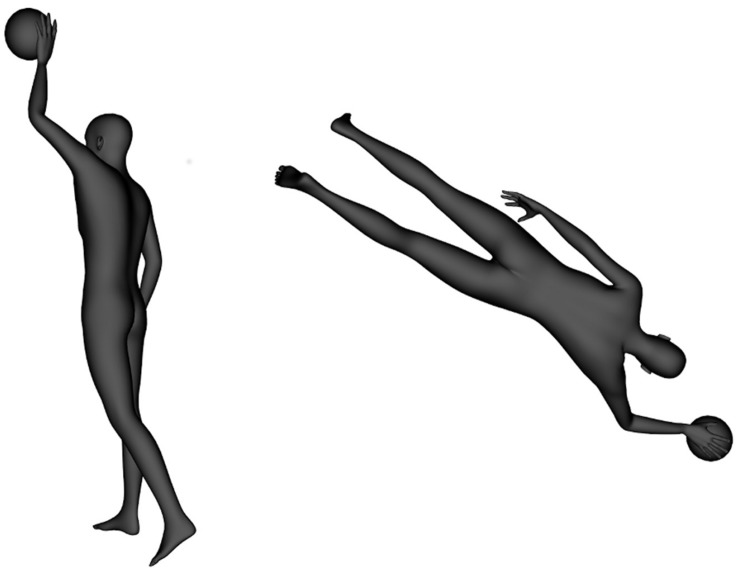
**Examples of two stimuli**. The left was rotated around the longitudinal axis (60°), the right around the depth axis (120°).

The stimuli are shown in **Figure 3**. When rotating around axes other than the depth axis, rotations of 90° lead to a partial occlusion of relevant cues. Therefore, the angle disparity was systematically varied in 60° steps and not in 45° steps, as in our two-dimensional experiments. The order of stimulus presentation was randomized for each participant. Participants had to decide as quickly and correctly as possible whether a ball is held in the right or left hand.

#### Data Analysis

To test whether elite team sports athletes outperform recreational athletes in the process of encoding/giving a motor response, an independent-samples *t*-test was conducted for response times at the angle disparity of 0°. A repeated-measures ANOVA of all angles >0° was conducted to analyze group differences in the process of mental rotation. Response times based on wrong answers were not analyzed.

### Results

**FIGURE 4:**
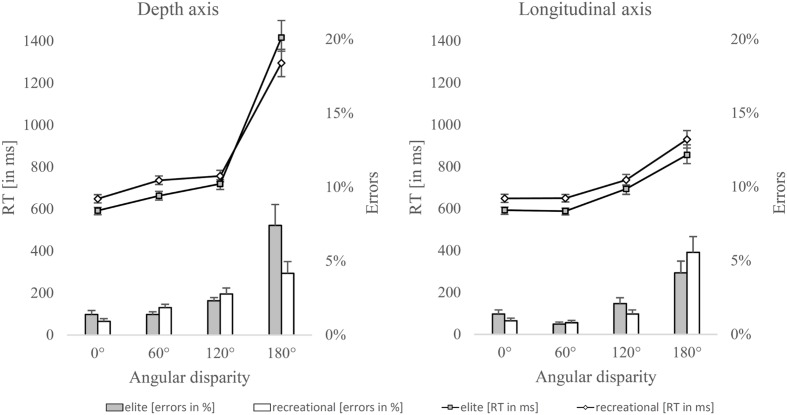
**Response times and error rates for stimuli rotated around depth (left side) and longitudinal axis (right side)**. Error bars represent standard errors.

The results are shown separately for both axes. **Figure 4** shows response times when stimuli were rotated around the depth axis and the longitudinal axis.

An independent-sample *t*-test showed a statistically significant difference in response times at an angle disparity of 0° between elite (*M* = 592.5, *SD* = 77.8) and recreational athletes (*M* = 648.9, *SD* = 101.9), *p* = 0.026, 95% CI [0.076, 1.168], *d* = 0.62.

A repeated measures ANOVA with factors disparity (60°, 120°, and 180°), axis (depth and longitudinal) and group (elite and recreational athletes) was used to determine group differences in mental rotation. The main effect of group was not significant, *F*(1,52) = 0.537, *p* = 0.467, and $\eta_{p}^{2}$ = 0.010. The interactions between disparity and group (*F*(2,104) = 1.709, *p* = 0.186, and $\eta_{p}^{2}$ = 0.032) and between axis and group were not significant (*F*(1,52) = 3.447, *p* = 0.069, and $\eta_{p}^{2}$ = 0.062) either. A significant 3-way interaction was found between axis, disparity and group (*F*(2,104) = 4.320, *p* = 0.032, and $\eta_{p}^{2}$ = 0.077), which can be assumed to be grounded on the opposite tendency in response times at 60° and 180° when rotated around the depth axis. *Post hoc* tests reveal statistically significant differences at 60° for rotations around the longitudinal axis (elite athletes: *M* = 588.5 ms, *SD* = 77.8; recreational athletes: *M* = 648.8 ms, *SD* = 93.2; *p* = 0.010, 95% CI [0.143, 1.242], *d* = 0.70) and for the depth axis (elite athletes: *M* = 662.6 ms, *SD* = 111.4; recreational athletes: *M* = 737.0 ms, *SD* = 104.9; *p* = 0.015, 95% CI [0.145, 1.243], *d* = 0.69.

### Discussion

In this experiment, we found no group differences in the process of mental rotation, even when controlling for gender differences and raising the level of expertise. However, our results are consistent with the findings of Jansen et al. (2012), who compared soccer players to non-athletes and found no difference in mental rotation. In one study that did show differences, differences were only found between athletes and non-athletes, but not between athletes from open- and closed-skill sports (Ozel et al., 2004). In our study, the control group was also physically active, which could explain why we observed no differences in the process of mental rotation.

However, when we look at the 0° condition, we see some differences. Elite athletes are responding faster in a 2-choice reaction time test (measured at 0° angle disparity), but not in mental rotation. This corroborates the observations by Jansen et al. (2012) that team sports athletes outperform controls concerning choice response time, but not in mental rotation. The trend that athletes perform better when responding to human figures in 0° position is probably not related to athletes simply having higher motor speed. There are findings that speak for a faster signal transmission in visual pathways, leading to shorter visual evoked potential latencies and shorter response times in volleyball players compared to controls (Zwierko et al., 2010). The differences in response times at 60° can be ascribed to the same cause, because there is no expert advantage at higher angle disparities.

Both experiments 1 and 2 did not reveal an expert advantage in mental rotation performance, but Experiment 2 revealed that experts in team sports showed better performance in 2-choice reaction time tests. Because a previous study showed that ex-Gaussian parameters in choice response tasks are related to working memory, reasoning and information processing speed (Schmiedek et al., 2007), we specifically investigated two of these cognitive skills and others in Experiment 3.

## Experiment 3: Basic Cognitive Skills of Elite and Recreational Athletes

Experiment 3 investigated whether elite athletes differ from recreational athletes in basic cognitive skills such as information processing speed, attention, letter readout speed, and memory span. If there is a cognitive skill transfer from frequent competitive training on cognition, both groups should differ in the non-sports-specific cognitive skills. The results by Voss et al. (2010) speak for a better performance of athletes in varied attention paradigms and in processing speed. We hypothesized a significant difference between both groups regarding the basic cognitive measures with advantages for the experts.

### Materials and Methods

#### Participants

In this experiment, elite athletes (*n* = 26, 13 females) playing soccer or volleyball were compared to recreational athletes (*n* = 26, 11 females). Mean age in the elite-athletes group was 21.9 years (*SD* = 3.81, range 17–32) and in the recreational sports group 22.0 years (*SD* = 3.15, range 18–29). The elite athletes consisted of soccer players of a 3rd-national-league team and volleyball players playing in the first German division.

#### Cognitive Tests

Sustained attention was measured by the d2-R test, which measures concentration and focused attention and requires the selection of relevant stimuli under time pressure (Brickenkamp et al., 2010). A test sheet contains 14 lines with 47 characters each. Each line consists of a random sequence of lowercase letters ‘*p*’ and ‘*d*’. The single characters are each framed with none, one, or two vertical dashes at top and/or bottom. The task of the participant is to strike out all lowercase ‘*d*’ letters which are surrounded by a total of just two vertical dashes (top and bottom combined). The other characters are distractors and must be ignored. Every 20 s, the examiner instructs the participants to move to the next line. During the evaluation of *d*2-*R* tests, three components are evaluated: the concentration ability (CA), the processed target objects (PTO) and the error rates. The CA value is described as most resistant against strategies that emphasize either speed or accuracy, because it takes PTO and errors into account (Brickenkamp et al., 2010). It is considered the best measure of the concentration ability while the PTO-value rather represents a measure of the pace of work, which should not have been primarily measured by this test.

Processing speed was measured by the ZVT (“Zahlen-Verbindungs-Test”; Oswald and Roth, 1987), a German equivalent to the Trail Making Test A (Reitan, 1958). Trail-making tasks place demands on visual search, speed of processing, and divided attention (Rizzo et al., 2000). The numbers 1–90 are presented in a 10 × 9 matrix and have to be connected in ascending order with a pen. The next higher number is always located in an adjacent position. Each participant solves four tests. The dependent variable is the arithmetic mean of the processing times.

As another measure of processing speed and as a measure of the memory span, the KAI-N (short test of general intelligence) (Lehrl and Blaha, 2001) was used. The test consists of two subtests, letter readout speed and memory span. In the subtest which measures letter readout time, 20 disjointed letters are printed in random order on a DIN A5 large card. This line must be read in a low voice by the subject as quickly as possible. The examiner stops the time required and the test is performed four more times with different letter sequences. The shortest reading time is noted and is the raw value for the calculation of the speed of information processing. Due to the short duration, the test requires fast perception of the presented letters to start quickly with maximum reading speed. Memory span is measured with the subtest character repetition. At the beginning, the examiner reads a sequence of numbers with three digits with a one second pause in between. This number sequence is extended stepwise if the numbers are repeated successfully. It is recorded how many numbers can be correctly reproduced. The same procedure is carried out with letters.

### Results

**FIGURE 5:**
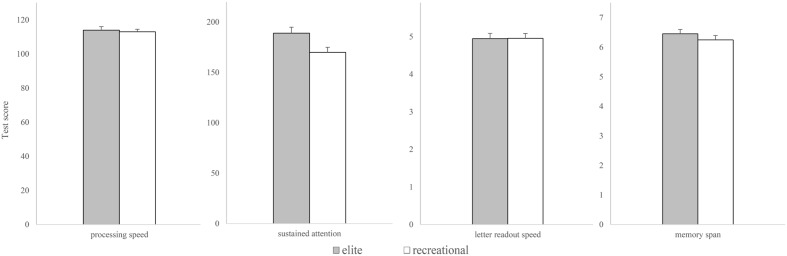
**Performance in cognitive tests of processing speed, sustained attention, memory span, and letter readout speed**. Error bars represent standard errors.

An overview about the results of both groups in the cognitive tests is shown in **Figure 5**. A MANOVA with four cognitive tests and two groups showed a significant main effect of group, *F*(4,45) = 3.534, *p* = 0.014, Wilk’s Λ = 0.761, $\eta_{p}^{2}$ = 0.239. Power to detect the effect was .828. *Post hoc* tests reveal a significant difference between elite (*M* = 189.44, *SD* = 30.17) and recreational (*M* = 170.35, *SD* = 25.12) athletes in sustained attention, *t*(49) = 2.46, *p* = 0.018, 95%, CI [0.128, 1.247], *d* = 0.69. The groups did not differ in other cognitive tests (all *p*-values > 0.3).

### Discussion

The results suggest that elite athletes have an advantage over recreational athletes in sustained attention, which is contradictory to findings by Memmert et al. (2009), who found no difference between experts from team sports, non-team sport athletes, and novices in three attention tasks. However, other studies found that elite athletes do perform better on attention tasks (Alves et al., 2013; Verburgh et al., 2014). The small-to-medium effect size for the group difference seen in our results matches the effect size for varied attention paradigms in the meta-analysis by Voss et al. (2010).

While our previous experiments only showed advantages for elite athletes compared to recreational athletes in choice response (the response time at non-rotated stimuli in a mental rotation task) times and sustained attention, they did not show these advantages in mental rotation and other non-sports-specific cognitive measures. To investigate the underlying cognitive skills involved in mental rotation, in Experiment 4 we took the same recreational athletes (control) group from Experiment 3 and tested their mental rotation performance. Then we combined this data with that from Experiment 3 to analyze the relationship of these aspects (basic cognitive skills and mental rotation).

## Experiment 4: Analyzing the Relationship Between Mental Rotation and Basic Cognitive Measures

The previous experiments, with elite and recreational athletes as participants, have shown that expertise cannot explain a significant amount of variance in cognitive skills, except in sustained attention. Cognitive skills such as working memory or attention are often associated with spatial abilities (Miyake et al., 2001). Thus, here we used an approach to investigate the relationship between basic cognitive measures and mental-rotation response times, which represents a promising way to investigate which basic cognitive measures underlie mental rotation performance.

Most studies on this relationship have been conducted with working memory paradigms. Working memory capacity was found to play a role when solving mental-rotation tasks (Kaufman, 2007; Colom et al., 2008; Pardo-Vazquez and Fernandez-Rey, 2012). A modified mental-rotation task, which puts higher demand on working-memory capacity, revealed decreased mental-rotation speed during the retention phase of a visual working-memory task when alphanumeric characters had to be judged in a mirror-normal task. This indicates that keeping information stored in the memory is important for mental rotation. The working-memory tasks differed in the load of object memory or spatial memory. The only rotation-dependent effects on rotation speed that were shown in the case of the working-memory task required the storage of objects, but not spatial information (Hyun and Luck, 2007). There is also neuroscientific evidence that working memory is used when solving mental-rotation tasks (Prime and Jolicoeur, 2010), which means that mental rotation involves representations which are maintained in visual short-term memory. In another dual-task paradigm, negative interferences between visuospatial attention and a mental rotation task were shown (Pannebakker et al., 2011).

There is also evidence that mental rotation performance correlates with processing speed, measured by a trail-making test (Jansen et al., 2013). These findings led to our assumption that all cognitive measures correlate with mental-rotation performance, measured by response times at different angle disparities. Thus, the aim of Experiment 4 is to determine whether there is a relationship between basic cognitive measures and mental rotation. Therefore, we set a focus on response times at the angle disparity of 180°.

### Materials and Methods

#### Participants

The recreational athletes (*n* = 26, 11 females) participating in Experiment 3 were also tested in a mental rotation experiment. Their mean age was 22.0 years (*SD* = 3.15, range 18–29).

#### Apparatus and Stimuli

**FIGURE 6:**
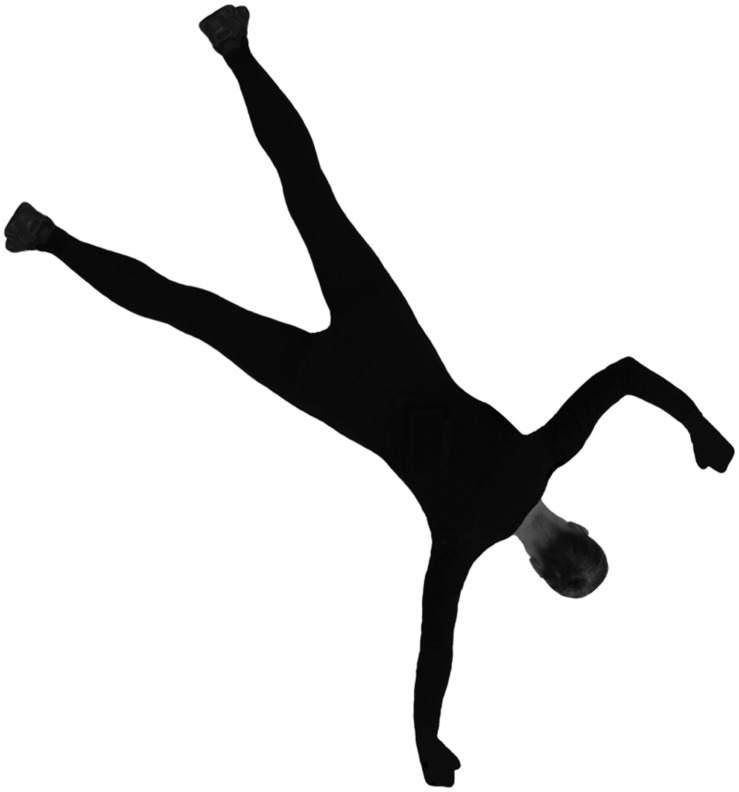
**Experiment 4**. Human figure at 135° angle disparity with left arm bent.

As a measure of mental rotation, we used a human stimuli in back view rotated around the depth axis in 45° steps (**Figure 6**). Participants had to decide as quickly and accurately as possible which arm is bent.

### Dependent Variables and Statistical Analysis

The response time was calculated as the difference between the occurrence of the stimulus and the motor response. This variable includes all the processes of perception and identification of the object, mental rotation of the object, and motor execution. Furthermore, the percentage of incorrect responses was measured. Incorrect responses were not included in further analyses.

To explore the contribution of cognitive measures to mental-rotation performance, a multiple linear regression analysis with five blocks (RT at 0°, 45°, 90°, 135°, and 180°) and four predictors (attention, memory span, letter readout speed, and processing speed) was computed. The Pearson product correlations are also reported to give a detailed view over the data. The analysis focuses on the extreme points of angle disparities, especially on response times at 180° angle disparity, because they contain the largest share of the process of mental rotation. For the angle disparity of 180°, an analysis of standard residuals showed that the data contained no outliers (Std. Residual Min = -2.07, Std. Residual Max = 2.02). The data met the assumption of independent errors (Durbin–Watson value = 2.275). Tests to see if the data met the assumption of collinearity indicated that multicollinearity was not a concern (attention, Tolerance = 0.51, *VIF* = 1.95; letter readout speed, Tolerance = 0.85, *VIF* = 1.18; memory span, Tolerance = 0.83, *VIF* = 1.21; processing speed, Tolerance = 0.60, *VIF* = 1.66). The histogram of standardized residuals indicated that the data contained approximately normally distributed errors. This also applies to the normal P–P plot of standardized residuals, which showed points that were not completely on the line, but close. The scatterplot of standardized residuals speaks for homogeneity of variance and linearity of the data. Results concerning the other angle disparities are also reported as well.

### Results

**FIGURE 7:**
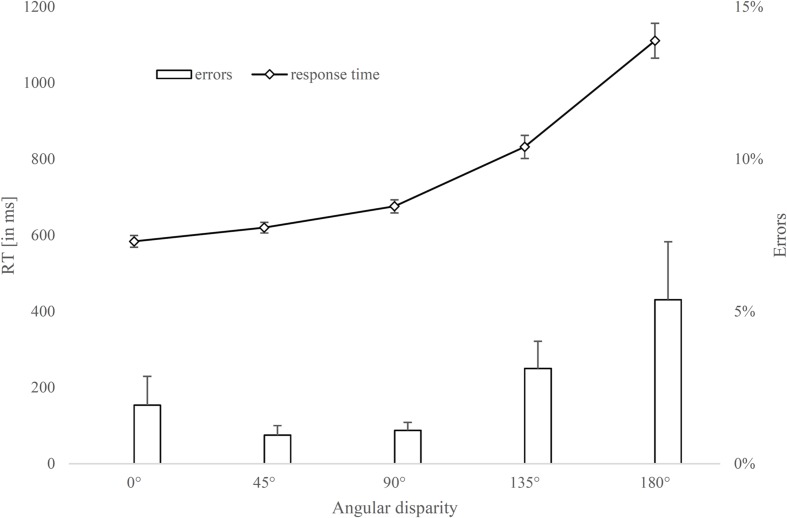
**Response times and error rates for a two-dimensional human figure rotated around the depth axis for each angle disparity**. Error bars represent standard errors.

Response times and error rates of the two-dimensional human stimulus are shown in **Figure 7**.

#### Pearson Correlations between Mental Rotation Response Times and Cognitive Measures

The Pearson product correlations between response times of a human stimulus at each angle disparity and predictor variables (basic cognitive measures) are presented in **Table 1**. As expected, the response times at different angles correlate highly with each other. At high angle disparities, processing speed (as measured by the ZVT) shows the highest correlations, whereas letter readout time shows highest correlations with response times at 0° angle disparity.

**Table 1:** Pearson product correlations between scores in basic cognitive measures and response times in the mental rotation test.

	1	2	3	4	0°	45°	90°	135°	180°
1. Sustained attention	1								
2. Letter readout time	-0.34	1							
3. Memory span	-0.25	-0.11	1						
4. Processing speed	0.59*^∗^*	-0.23	0.07	1					
0°	-0.23	0.56*^∗^*	-0.16	-0.29	1				
45°	-0.14	0.34	-0.30	-0.19	0.82*^∗^*	1			
90°	-0.27	0.20	-0.22	-0.35	0.60*^∗^*	0.80*^∗^*	1		
135°	-0.31	0.32	-0.26	-0.41*^∗^*	0.67*^∗^*	0.85*^∗^*	0.89*^∗^*	1	
180°	-0.32	0.42*^∗^*	-0.23	-0.53*^∗^*	0.67*^∗^*	0.75*^∗^*	0.77*^∗^*	0.85*^∗^*	1

**N* = 26. ^∗^*p* < 0.05*.

#### Linear Regression between Mental Rotation Response Times and Cognitive Measures

The multiple linear regression analysis was calculated to predict response times at a certain angle disparity (0–180°) based on the cognitive measures. Using the enter method, it was found that cognitive measures only explain a significant amount of the variance in response times at an angle disparity of 180° (*F*(4, 21) = 3.50, *p* = 0.024, *R*^2^ = 0.40, *R^2^_adjusted_* = 0.29). To evaluate how the four cognitive measures contribute individually to the result, the standardized coefficients (β) and the standard errors are reported in **Table 2**. Most important, processing speed (via the ZVT score) explains the largest share of variance in response times at 180°, β = -0.45, *t*(21) = 2.08, *p* = 0.05. But there are also interesting findings in the response time at 0°, indicating a significant share of variance explanation by letter readout time, β = 0.52, *t*(21) = 2.70, *p* = 0.01.

**Table 2:** Multiple linear regression between response times of stimulus ‘human figure’ and cognitive measures.

	Angular disparity									
	0°		45°		90°		135°		180°	
Predictor	β	*SE* β	β	*SE* β	β	*SE* β	β	*SE* β	β	*SE* β
Sustained attention	0.04	0.25	-0.08	0.27	-0.18	0.28	-0.17	0.26	0.01	0.24
Letter readout time	0.52	0.19	0.26	0.21	0.06	0.21	0.18	0.20	0.30	0.18
Memory span	-0.08	0.19	-0.26	0.22	-0.24	0.22	-0.26	0.20	-0.16	0.19
Processing speed	-0.18	0.23	-0.05	0.25	-0.20	0.25	-0.25	0.24	-0.45	0.22
Total *R*^2^	0.346		0.196		0.185		0.278		0.400	
Adj. *R*^2^	0.222		0.043		0.030		0.141		0.285	

### Discussion

The purpose of Experiment 4 was to evaluate the relationship between mental rotation performance and basic cognitive measures. The results of this study provide further answers to the question of which cognitive processes are involved when solving a chronometric mental-rotation task. Results of the linear regression analysis suggest there is an overlap between cognitive measures and mental rotation performance. Similar to a previous finding (Jansen et al., 2013), we found a medium-sized relationship between processing speed (measured by time required in a trail-making test) and mental rotation performance. When presenting human figures at 180°, response times contain a larger share of the process by which participants must rotate the figure into the 0° position. At 180°, processing speed (β = -0.45) seems to play a more important role than the other cognitive measures, but letter readout time also explains a fair amount of variance (β = 0.30). Both of the measures which explain most of the variance in mental rotation are speed related. Therefore, it can be assumed that speed-related measures are important for mental rotation. Sustained attention (β = 0.01) and memory span (β = -0.16) show low correlations with response times at 180°, so they do not play a substantial role for mental rotation performance. However, letter readout time shows medium-sized correlations (β = 0.52) with the response time at 0°, which speaks for an overlap between this component and the encoding/giving a motor response process.

## General Discussion

This series of experiments evaluated whether elite athletes from team sports perform better at non-sports-specific tasks in the laboratory, i.e., on visuospatial and basic cognitive tests. Observed differences can be based on the fact that cognitive skills trained in team sports might induce a transfer to performance in these tests. However, these differences can also be caused by genetic predispositions in these cognitive skills. In three experiments, elite athletes in team sports were compared to recreational athletes in mental rotation, in choice response times and in basic cognitive measures. In the fourth experiment, we investigated how much variance in mental rotation response times is explained by basic cognitive measures. As far as the authors know, the approach of examining the relationship between response times in mental rotation and different basic cognitive measures to investigate their share of variance has not been done before.

In Experiment 1, we compared elite female soccer players to recreational athletes in mental rotation of two-dimensional human figures and found that mental rotation and 2-choice response times were not affected by the level of expertise. In Experiment 2, we used three-dimensional human stimuli which increased the difficulty of the task. Furthermore, we tested elite athletes that had a higher level of expertise. We replicated the finding concerning the process of mental rotation in this experiment (i.e., no differences), but found advantages in 2-choice response times for the elite athletes. In the processes of encoding and giving a motor response, measured by response times at small angle disparities, we found an expertise advantage. Whether differences in response times are based on higher motor speed of experts or on other processes like perception and encoding cannot be determined from behavioral data in this experiment. Measurement of simple response times and other choice reaction tests could address this issue in further studies, as well as electrophysiological measures.

In Experiment 3, instead of testing visuospatial skills and choice response times, we examined elite athletes in basic cognitive skills. The results showed a statistically significant difference and a medium-sized effect in sustained attention in favor of elite athletes. This is contradictory to findings by Memmert et al. (2009), but other studies support our findings (Vestberg et al., 2012; Alves et al., 2013; Verburgh et al., 2014). There were no differences in other basic cognitive measures. The heterogeneity in findings in different experiments on this issue could be related to the difference of expertise levels between the elite and control groups or to the differences in sport types. In a study which tested basketball, volleyball and water-polo players in a battery of cognitive tests, results showed that the experts outperformed novices only on selective tasks of the battery (Kioumourtzoglou et al., 1998). According to the meta-analysis by Voss et al. (2010), all players in these groups belong to the group of strategic sports. The kind of tasks which showed elite advantages were dependent on sports type and most of the differences only showed up in sports-specific cognitive tests. Voss et al. (2010) report evidence for better performance in varied attention paradigms and in processing speed of elite athletes, but also studies that speak against expert advantages in basic cognitive measures. The appearance of these expertise effects could depend on the exact type of tasks. Speculating, our findings in Experiment 3 could have implications for talent identification and training objectives for athletes.

The supplementary analysis in Experiment 4 connects the mental rotation experiments to basic cognitive measures. According to a previous finding (Jansen et al., 2013), expertise was shown not to be a good predictor for performance in mental rotation. Thus, Experiment 4 focused on the underlying processes in mental rotation, we found that there is a medium-sized relationship between mental rotation performance (measured at 180° angle disparity) and processing speed (measured by a trail-making test). This may be because the mental processing of rotating a stimulus shows overlaps to the basic cognitive measure of processing speed. The share of variance could also be caused by a superordinate factor, like intelligence, which is assumed to be correlated to both mental rotation and processing speed. For response times at 0°, which can be viewed as a 2-choice response time, letter readout speed is by far the best predictor and shows a medium-sized effect. It can be speculated that this might have to do with the similarity in tasks. In choice response tasks in this experiment, a fast perception of the stimulus is mandatory to give a maximally fast motor response with the correct index finger. In the letter readout test, the 20 presented letters have to be perceived quickly to start with the verbal response and a tight continuous sensorimotor coupling is required to solve the task in minimal time. This overlap in tasks could explain the share of variance. Why is it that we did not find a stronger correlation between memory span (measured by digit und letter span) and mental rotation? In the current experiment, we used a verbal memory span test. However, some studies show that the working memory is domain specific (i.e., verbal and spatial). For example, a reading span test is a good predictor for performance in a verbal Scholastic Aptitude Test (SAT), but performance in spatial tasks could not significantly predict the verbal SAT performance. However, performing two spatial working memory tasks could predict achievements in various visuospatial tests; the achievements could not be predicted by the verbal working memory task, which speaks for a narrow transfer within a domain between cognitive skills (Shah and Miyake, 1996). Other findings show a correlation between mental rotation and spatial working memory, but not verbal working memory (Christie et al., 2013). Further studies using the approach should use tests of different domains of working memory to investigate the share of variance. Despite the duration of the mental rotation experiment of more than 10 minutes, which can be assumed to place demands on sustained attention, we found no relevant correlations between sustained attention and response times. Possibly mental rotation and the *d*2 - *R* test require different aspects of attention. In *d*2 - *R*, attention has to be sustained over a period of 280 s. The mental rotation experiments took longer, but had inter-stimulus intervals of 2 s in between, which requires a more wave-like attention curve.

There are several limitations in this series of studies. In future studies, expertise should no longer be treated as a categorical, but as a continuous variable, as Swann et al. (2015) suggested in their review. Athletes can be rated by the highest league in which they are or were playing, their rank on their team, their years of experience, and other factors. But also the competiveness of sports on the national and international level is a factor which should be included to calculate a score that can reflect the athletes’ level of expertise. This procedure would allow a more exact classification of expertise and enable other approaches to analyze the data. When analyzing choice response times from mental rotation experiments, it would be very helpful to also test single reaction times and classical choice response times without the complications of rotational uncertainty. This would allow to give a clearer answer to the question why participants are faster at non-rotated stimuli.

As a result of conducting this series of studies, there are three main conclusions: First, especially in the rather conservative approach to use recreational athletes and not non-athletes as a control group, the level of expertise of the elite athletes should be as high as possible to cover a large spectrum of the expertise continuum. Second, the finding that elite athletes perform better in a basic cognitive test of sustained attention indicates that this skill seems relevant for team sports. Third, elite athletes had shorter response times in human stimuli at 0 and 60° angle disparity. Future studies will investigate if this finding is based on better coping with rotational uncertainty, faster encoding or faster motor responses in each athlete. Fourth, processing speed seems to play an important role in predicting mental rotation times. In future mental rotation studies, processing speed should be considered to be taken as a control variable. It could also be used to counter-balance experimental groups in mental rotation experiments.

## Author Contributions

ZE conceived the project and supervised the experiments. HH, ZE and AK designed the experiments. HH and ZE developed the stimuli. HH, AK and MF performed the experiments. HH, AK and ZE analyzed and interpreted the data. HH prepared the manuscript. ZE, AK, and MF edited the manuscript. All authors approved the final version to be published and are accountable for all aspects of the work.

## Conflict of Interest Statement

The authors declare that the research was conducted in the absence of any commercial or financial relationships that could be construed as a potential conflict of interest.

## References

[B1] Akarsu S. Çalişcan E. Dane Ş. (2009). Athletes have faster eye-hand visual reaction times and higher scores on visuospatial intelligence than nonathletes. *Turk. J. Med. Sci.* 39 871–874.

[B2] Alves H. Voss M. W. Boot W. R. Deslandes A. Cossich V. Salles J. I. (2013). Perceptual-cognitive expertise in elite volleyball players. *Front. Psychol.* 4:36 10.3389/fpsyg.2013.00036 PMC3590639 23471100

[B3] Brickenkamp R. Schmidt-Atzert L. Liepmann D. (2010). *Test d2 - Revision: D2-R; Aufmerksamkeits- und Konzentrationstest*. Göttingen: Hogrefe.

[B4] Broadbent D. P. Causer J. Williams A. M. Ford P. R. (2015). Perceptual-cognitive skill training and its transfer to expert performance in the field: future research directions. *Eur. J. Sport Sci.* 15 322–331. 10.1080/17461391.2014.957727 25252156

[B5] Christie G. J. Cook C. M. Ward B. J. Tata M. S. Sutherland J. Sutherland R. J. (2013). Mental rotational ability is correlated with spatial but not verbal working memory performance and P300 amplitude in males. *PLoS ONE* 8:e57390 10.1371/journal.pone.0057390 PMC3577737 23437381

[B6] Colcombe S. Kramer A. F. (2003). Fitness effects on the cognitive function of older adults: a meta-analytic study. *Psychol. Sci.* 14 125–130. 10.1111/1467-9280.t01-1-01430 12661673

[B7] Colom R. Abad F. J. Quiroga M. Á Shih P. C. Flores-Mendoza C. (2008). Working memory and intelligence are highly related constructs, but why? *Intelligence* 36 584–606. 10.1016/j.intell.2008.01.002

[B8] Cooper L. A. (1975). Mental rotation of two-dimensional shapes. *Cogn. Psychol.* 7 20–43. 10.1016/0010-0285(75)90003-1

[B9] Cooper L. A. Shepard R. N. (1973). “Chronometric studies of the rotation of mental images,” in *Visual Information Processing*, ed. Chase W. G. (New York, NY: Academic Press), 75–176.

[B10] Dahlin E. Neely A. S. Larsson A. Bäckman L. Nyberg L. (2008). Transfer of learning after updating training mediated by the striatum. *Science* 320 1510–1512. 10.1126/science.1155466 18556560

[B11] Faubert J. (2013). Professional athletes have extraordinary skills for rapidly learning complex and neutral dynamic visual scenes. *Sci. Rep.* 3:1154 10.1038/srep01154 PMC3560394 23378899

[B12] Heirani A. VaziniTaher A. Soori Z. Rahmani M. (2012). Relationship between choice reaction time and expertise in team and individualsports: a gender differences approach. *Aust. J. Basic Appl. Sci.* 6 344–348.

[B13] Hillman C. H. Erickson K. I. Kramer A. F. (2008). Be smart, exercise your heart: exercise effects on brain and cognition. *Nat. Rev. Neurosci.* 9 58–65. 10.1038/nrn2298 18094706

[B14] Hyun J.-S. Luck S. J. (2007). Visual working memory as the substrate for mental rotation. *Psychon. B Rev.* 14 154–158. 10.3758/BF03194043 17546746

[B15] Ilan A. B. Miller J. (1994). A violation of pure insertion: mental rotation and choice reaction time. *J. Exp. Psychol.-Hum. Percept. Perform.* 20 520–536. 10.1037/0096-1523.20.3.520 8027713

[B16] Jaeggi S. M. Buschkuehl M. Jonides J. Perrig W. J. (2008). Improving fluid intelligence with training on working memory. *Proc. Natl. Acad. Sci. U.S.A.* 105 6829–6833. 10.1073/pnas.0801268105 18443283 PMC2383929

[B17] Jansen P. Kellner J. Rieder C. (2013). The improvement of mental rotation performance in second graders after creative dance training. *Creat. Educ.* 4 418–422. 10.4236/ce.2013.46060

[B18] Jansen P. Lehmann J. (2013). Mental rotation performance in soccer players and gymnasts in an object-based mental rotation task. *Adv. Cogn. Psychol.* 9 92–98. 10.2478/v10053-008-0135-8 23833695 PMC3700661

[B19] Jansen P. Lehmann J. van Doren J. (2012). Mental rotation performance in male soccer players. *PLoS ONE* 7:e48620 10.1371/journal.pone.0048620 PMC3484043 23119073

[B20] Jansen-Osmann P. Heil M. (2007). Suitable stimuli to obtain (no) gender differences in the speed of cognitive processes involved in mental rotation. *Brain Cogn.* 64 217–227. 10.1016/j.bandc.2007.03.002 17433514

[B21] Jola C. Mast F. W. (2005). Mental object rotation and egocentric body transformation: two dissociable processes? *Spat. Cogn. Comput.* 5 217–237.

[B22] Karbach J. Verhaeghen P. (2014). Making working memory work: a meta-analysis of executive-control and working memory training in older adults. *Psychol. Sci.* 25 2027–2037. 10.1177/0956797614548725 25298292 PMC4381540

[B23] Kaufman S. B. (2007). Sex differences in mental rotation and spatial visualization ability: can they be accounted for by differences in working memory capacity? *Intelligence* 35 211–223. 10.1016/j.intell.2006.07.009

[B24] Kioumourtzoglou E. Kourtessis T. Michalopoulou M. Derri V. (1998). Differences in several perceptual abilities between experts and novices in basketball, volleyball and water-polo. *Percept. Mot. Skills* 86 899–912. 10.2466/pms.1998.86.3.899 9656285

[B25] Kosslyn S. M. Digirolamo G. J. Thompson W. L. Alpert N. M. (1998). Mental rotation of objects versus hands: neural mechanisms revealed by positron emission tomography. *Psychophysiology* 35 151–161. 10.1111/1469-8986.3520151 9529941

[B26] Lehrl S. Blaha L. (2001). *Messung des Arbeits-Gedächtnisses KAI-N*. Ebersberg: Vless.

[B27] Linn M. C. Petersen A. C. (1985). Emergence and characterization of sex differences in spatial ability: a meta-analysis. *Child Dev.* 56 1479–1498. 10.2307/1130467 4075870

[B28] Mann D. T. Y. Williams A. M. Ward P. Janelle C. M. (2007). Perceptual-cognitive expertise in sport: a meta-analysis. *J. Sport Exerc. Psychol.* 29 457–478. 17968048 10.1123/jsep.29.4.457

[B29] Marchetti R. Forte R. Borzacchini M. Vazou S. Tomporowski P. D. Pesce C. (2015). Physical and motor fitness, sport skills and executive function in adolescents: a moderated prediction model. *Psychology* 6 1915–1929. 10.4236/psych.2015.614189

[B30] Memmert D. Simons D. J. Grimme T. (2009). The relationship between visual attention and expertise in sports. *Psychol. Sport Exerc.* 10 146–151. 10.1016/j.psychsport.2008.06.002

[B31] Miyake A. Friedman N. P. Rettinger D. A. Shah P. Hegarty M. (2001). How are visuospatial working memory, executive functioning, and spatial abilities related? A latent-variable analysis. *J. Exp. Psychol. Gen.* 130 621–640. 10.1037/0096-3445.130.4.621 11757872

[B32] Moreau D. (2012). The role of motor processes in three-dimensional mental rotation: shaping cognitive processing via sensorimotor experience. *Learn. Indiv. Differ.* 22 354–359. 10.1016/j.lindif.2012.02.003

[B33] Moreau D. Clerc J. Mansy-Dannay A. Guerrien A. (2012). Enhancing spatial ability through sport practice. *J. Ind. Diff.* 33 83–88. 10.1027/1614-0001/a000075

[B34] Oswald W. D. Roth E. (1987). *Der Zahlen-Verbindungs-Test (ZVT). Ein sprachfreier Intelligenz-Test zur Messung der “kognitiven Leistungsgeschwindigkeit”*. Göttingen: Hogrefe.

[B35] Ozel S. Larue J. Molinaro C. (2004). Relation between sport and spatial imagery: comparison of three groups of participants. *J. Psychol.* 138 49–64. 10.3200/JRLP.138.1.49-64 15098714

[B36] Pannebakker M. M. Jolicœur P. van Dam W. O. Band G. P. Ridderinkhof K. R. Hommel B. (2011). Mental rotation impairs attention shifting and short-term memory encoding: neurophysiological evidence against the response-selection bottleneck model of dual-task performance. *Neuropsychologia* 49 2985–2993. 10.1016/j.neuropsychologia.2011.06.021 21736889

[B37] Pardo-Vazquez J. L. Fernandez-Rey J. (2012). Working memory capacity and mental rotation: evidence for a domain-general view. *Span. J. Psychol.* 15 881–890. 10.5209/rev_SJOP.2012.v15.n3.39381 23156898

[B38] Parsons L. M. (1987). Imagined spatial transformations of one’s hands and feet. *Cogn. Psychol.* 19 178–241. 10.1016/0010-0285(87)90011-9 3581757

[B39] Perkins D. N. Salomon G. (1989). Are cognitive skills context bound. *Educ. Res.* 18 16–25. 10.3102/0013189X018001016

[B40] Peters M. (2005). Sex differences and the factor of time in solving Vandenberg and Kuse mental rotation problems. *Brain Cogn.* 57 176–184. 10.1016/j.bandc.2004.08.052 15708213

[B41] Piras A. Lobietti R. Squatrito S. (2014). Response time, visual search strategy, and anticipatory skills in volleyball players. *J. Ophthalmol.* 4 1–10. 10.1155/2014/189268 PMC4021845 24876946

[B42] Prime D. J. Jolicoeur P. (2010). Mental rotation requires visual short-term memory: evidence from human electric cortical activity. *J. Cogn. Neurosci.* 22 2437–2446. 10.1162/jocn.2009.21337 19702462

[B43] Raab M. (2014). SMART-ER: a situation model of anticipated response consequences in tactical decisions in skill acquisition - extended and revised. *Front. Psychol.* 5:1533 10.3389/fpsyg.2014.01533 PMC4285051 25610416

[B44] Reed C. L. Farah M. J. (1995). The psychological reality of the body schema: a test with normal participants. *J. Exp. Psychol. Hum. Percept. Perform.* 21 334–343. 10.1037/0096-1523.21.2.334 7714475

[B45] Reitan R. M. (1958). Validity of the trail making test as an indicator of organic brain damage. *Percept. Mot. Skills* 8 271–276. 10.2466/pms.1958.8.3.271

[B46] Rizzo M. Anderson S. W. Dawson J. Nawrot M. (2000). Vision and cognition in Alzheimer’s disease. *Neuropsychologia* 38 1157–1169. 10.1016/S0028-3932(00)00023-3 10838150

[B47] Romeas T. Faubert J. (2015). Soccer athletes are superior to non-athletes at perceiving soccer-specific and non-sport specific human biological motion. *Front. Psychol.* 6:1343 10.3389/fpsyg.2015.01343 PMC4558464 26388828

[B48] Ruthruff E. Miller J. (1995). Can mental rotation begin before perception finishes? *Mem. Cogn.* 23 408–424. 10.3758/BF03197243 7666755

[B49] Schmiedek F. Oberauer K. Wilhelm O. Süss H.-M. Wittmann W. W. (2007). Individual differences in components of reaction time distributions and their relations to working memory and intelligence. *J. Exp. Psychol. Gen.* 136 414–429. 10.1037/0096-3445.136.3.414 17696691

[B50] Shah P. Miyake A. (1996). The separability of working memory resources for spatial thinking and language processing: an individual differences approach. *J. Exp. Psychol. Gen.* 125 4–27. 10.1037/0096-3445.125.1.4 8851737

[B51] Shepard R. N. Metzler J. (1971). Mental rotation of three-dimensional objects. *Science* 171 701–703. 10.1126/science.171.3972.701 5540314

[B52] Starkes J. L. (1987). Skill in field hockey: the nature of the cognitive advantage. *J. Sport Psychol.* 9 146–160.

[B53] Steggemann Y. Engbert K. Weigelt M. (2011). Selective effects of motor expertise in mental body rotation tasks: comparing object-based and perspective transformations. *Brain Cogn.* 76 97–105. 10.1016/j.bandc.2011.02.013 21429647

[B54] Swann C. Moran A. Piggott D. (2015). Defining elite athletes: issues in the study of expert performance in sport psychology. *Psychol. Sport Exerc.* 16 3–14. 10.1016/j.psychsport.2014.07.004

[B55] Tang Y. Y. Posner M. I. (2009). Attention training and attention state training. *Trends Cogn. Sci.* 13 222–227. 10.1016/j.tics.2009.01.009 19375975

[B56] Uttal D. H. Meadow N. G. Tipton E. Hand L. L. Alden A. R. Warren C. (2013). The malleability of spatial skills: a meta-analysis of training studies. *Psychol. Bull.* 139 352–402. 10.1037/a0028446 22663761

[B57] Verburgh L. Scherder E. J. A. van Lange P. A. M. Oosterlaan J. Perales J. C. (2014). Executive functioning in highly talented soccer players. *PLoS ONE* 9:e91254 10.1371/journal.pone.0091254 PMC3954684 24632735

[B58] Vestberg T. Gustafson R. Maurex L. Ingvar M. Petrovic P. García A. V. (2012). Executive functions predict the success of top-soccer players. *PLoS ONE* 7:e34731 10.1371/journal.pone.0034731 PMC3319604 22496850

[B59] Voelcker-Rehage C. Godde B. Staudinger U. M. (2011). Cardiovascular and coordination training differentially improve cognitive performance and neural processing in older adults. *Front. Hum. Neurosci* 5:26 10.3389/fnhum.2011.00026 PMC3062100 21441997

[B60] Voss M. W. Kramer A. F. Basak C. Prakash R. S. Roberts B. (2010). Are expert athletes ‘expert’ in the cognitive laboratory? A meta-analytic review of cognition and sport expertise. *Appl. Cogn.Psychol.* 24 812–826. 10.1002/acp.1588

[B61] Voyer D. (2011). Time limits and gender differences on paper-and-pencil tests of mental rotation: a meta-analysis. *Psychon. B Rev.* 18 267–277. 10.3758/s13423-010-0042-0 21327340

[B62] Voyer D. Jansen P. (2015). Sex differences in chronometric mental rotation with human bodies. *Psychol. Res.* 10.1007/s00426-015-0701-x [Epub ahead of print]. 26358053

[B63] Wang C.-H. Tsai C.-L. Tu K.-C. Muggleton N. G. Juan C.-H. Liang W.-K. (2015). Modulation of brain oscillations during fundamental visuo-spatial processing: a comparison between female collegiate badminton players and sedentary controls. *Psychol. Sport Exerc.* 16 121–129. 10.1016/j.psychsport.2014.10.003

[B64] Wexler M. Kosslyn S. M. Berthoz A. (1998). Motor processes in mental rotation. *Cognition* 69 77–94. 10.1016/S0010-0277(98)00032-8 9775517

[B65] Willis S. L. Tennstedt S. L. Marsiske M. Ball K. Elias J. Koepke K. M. (2006). Long-term effects of cognitive training on everyday functional outcomes in older adults. *J. Am. Med. Assoc.* 296 2805–2814. 10.1001/jama.296.23.2805 PMC2910591 17179457

[B66] Zacks J. M. Vettel J. M. Michelon P. (2003). Imagined viewer and object rotations dissociated with event-related fMRI. *J. Cogn. Neurosci.* 15 1002–1018. 10.1162/089892903770007399 14614811

[B67] Zwierko T. Osinski W. Lubinski W. Czepita D. Florkiewicz B. (2010). Speed of visual sensorimotor processes and conductivity of visual pathway in volleyball players. *J. Hum. Kinet.* 23 21–27. 10.2478/v10078-010-0003-8

